# Temporal patterns of respiratory rate exposure and mortality in critically ill patients: a retrospective multi-database cohort study

**DOI:** 10.3389/fmed.2026.1900968

**Published:** 2026-09-30

**Authors:** Jingqing Xu, Min Li, Zhulin Wang, Linqian Jiang, Xiankun Lin, Xiuling Shang, Xincai Wang

**Affiliations:** 1 Department of Critical Care Medicine, Shengli Clinical Medical College of Fujian Medical University, Fuzhou University Affiliated Provincial Hospital, Fujian Provincial Hospital, Fujian Provincial Center for Critical Care Medicine, Fujian Provincial Key Laboratory of Critical Care Medicine, Fuzhou, China; 2 Department of Pediatric Intensive Care Unit, National Children’s Medical Center, Shanghai Children’s Medical Center, School of Medicine, Shanghai Jiao Tong University, Shanghai, China

**Keywords:** exposure duration, intensive care unit, mortality, respiratory rate, temporal patterns

## Abstract

**Objectives:**

To investigate the associations between respiratory rate (RR) exposure patterns—both intensity and duration—and mortality in critically ill patients.

**Methods:**

This retrospective cohort study analysed 160,075 adult ICU patients from three international databases (HiRID, MIMIC-IV, eICU). The primary analysis used a landmark design: RR exposure and all covariates were ascertained during the first 24 h after ICU admission, and outcome follow-up began at the 24-hour landmark. Heatmap visualisation examined RR intensity-duration patterns. Cox and logistic regression models related the proportion of monitoring time spent in each RR range to mortality, adjusted for demographics, disease severity, laboratory values, comorbidities, treatment limitations, ventilation and sedation. Restricted cubic splines assessed the dose-response relationship for time-weighted mean RR. Sensitivity analyses examined alternative exposure windows, competing risks, multiple imputation, hospital-level clustering and a harmonised in-hospital mortality outcome.

**Results:**

A greater proportion of time in the 12–20 breaths/min range was associated with lower 28-day mortality in MIMIC-IV (HR 0.988, 95% CI 0.987–0.989 per 1 percentage point) and lower in-hospital mortality in eICU (OR 0.991, 95% CI 0.990–0.991). Risk rose progressively across the tachypnoeic ranges (>35 breaths/min: HR 1.026, 95% CI 1.023–1.028). Spline analysis identified a nadir of risk at a time-weighted mean RR of 16.3 breaths/min, with a markedly asymmetric relationship (*P* for non-linearity <0.001): the adjusted hazard ratio reached 1.38 at 20 and 2.19 at 24 breaths/min but rose only modestly below the nadir. The bradypnoea-mortality association seen with whole-stay exposure (HR 1.029) was absent under the landmark design (HR 0.990) in both databases. Tachypnoeic associations were robust to stratification by ventilation and sedation, clustering and multiple imputation.

**Conclusions:**

Respiratory rate showed an asymmetric, non-linear relationship with mortality, with lowest risk at approximately 16–17 breaths/min. The cumulative burden of tachypnoea during the first 24 h was consistently associated with higher mortality across three databases and robust to controls for reverse causation, ventilation and sedation; the apparent association for bradypnoea was not, and likely reflects terminal-phase respiratory depression. Respiratory rate should be regarded as a monitoring and early-warning signal that integrates both magnitude and duration, rather than as a therapeutic target.

## Introduction

Respiratory rate (RR) is a fundamental vital sign in acute care settings, yet it has long been recognized as “the neglected vital sign” despite its prognostic significance (1, 2). Mounting evidence demonstrates that RR abnormalities are strongly associated with adverse outcomes in hospitalized patients. Fieselmann et al. reported that the occurrence of one or more respiratory rates exceeding 27 breaths per minute within the preceding 72 h predicted in-hospital cardiopulmonary arrest, with a sensitivity of 0.54 and specificity of 0.83 (3). Respiratory rate has been incorporated into widely used early warning scores including the Modified Early Warning Score (MEWS) (4), National Early Warning Score (NEWS) (5), and quick Sequential Organ Failure Assessment (qSOFA) (6, 7). RR is a strong predictor and an important component in early warning models for cardiac arrest and ICU admission (5, 8). Respiratory rate variability (RRV) has also emerged as a prognostic marker, with investigations showing that RRV changes in the measurements preceding ICU transfer may predict clinical deterioration (9), while decreased RRV in critically ill populations has been associated with increased mortality (10). However, substantial heterogeneity exists in RR recording practices and measurement quality across studies (11, 12), limiting the reproducibility and interpretability of existing evidence.

A critical limitation of previous research is its focus on RR intensity (RR levels) while neglecting temporal patterns. Clinical observations suggest that patients with similar average respiratory rates may exhibit markedly different outcomes, potentially reflecting differences in the temporal dynamics of RR abnormalities. From a physiological perspective, it is biologically plausible that sustained tachypnea may lead to progressive respiratory muscle fatigue, increased work of breathing, and cumulative metabolic derangement, whereas intermittent episodes may allow for partial physiological recovery between exacerbations. However, most existing studies rely on intermittent measurements with variable recording compliance and observation frequency (13, 14), which cannot distinguish between sustained and intermittent patterns of RR derangement. Even investigations of respiratory rate variability have been limited by temporal resolution constraints in intermittently charted data (9), although decreased RRV in critically ill populations has been associated with increased mortality (10). This highlights two critical yet insufficiently characterized dimensions: continuous exposure duration (reflecting sustained physiological stress from uninterrupted RR abnormalities) and cumulative exposure proportion (indicating the overall temporal burden of RR derangement). The integration of both temporal dimensions—how long patients maintain abnormal RR values and what proportion of monitoring time is spent in different RR ranges—may provide more comprehensive prognostic insights than instantaneous measurements or simple time-averaged values alone.

The aim of this study is to systematically investigate the associations between respiratory rate exposure characteristics and clinical outcomes across both temporal dimensions. Specifically, we seek to identify RR ranges associated with lowest risk, characterize dose-response relationships between RR time-intensity exposure and outcomes, and inform respiratory monitoring strategies in intensive care units.

## Methods

### Study design

This retrospective cohort study examined associations between respiratory rate (RR) exposure patterns and mortality in critically ill patients using three complementary analytical approaches across multiple ICU databases. Time zero was defined as the time of admission to the index ICU stay. In the primary analysis, RR exposure was measured during a fixed 24-hour window beginning at time zero, and outcome follow-up began at the end of that window (the 24-hour landmark), so that the exposure window and the follow-up period do not overlap. All covariates were ascertained within the same 24-hour window. Analyses using exposure accumulated over the entire ICU stay are reported as sensitivity analyses.

### Data sources

Three databases were utilised. The HiRID database (Bern University Hospital, Switzerland) provides high-resolution, minute-level physiological monitoring data from critically ill patients, making it suitable for analysing the continuity of RR exposure (15). We used HiRID for the intensity-duration heatmap analysis with in-hospital mortality as the outcome. The MIMIC-IV database (Beth Israel Deaconess Medical Center, United States) contains comprehensive electronic health records from critically ill patients (16). Given its large sample size and detailed clinical information, MIMIC-IV was used for the primary landmark analysis of range-specific exposure and for the restricted cubic spline analysis, with 28-day mortality as the outcome. The eICU database (multiregional US institutions) was used to assess the transferability of these associations to an independent population, with in-hospital mortality as the outcome (17). Because the outcome definitions and the modelling approaches necessarily differ between databases, this analysis is presented as an assessment of transferability under differing outcomes rather than as external validation of a fixed prediction model. Data were extracted using SQL and Python 3.13.

### Inclusion and exclusion criteria

Inclusion criteria were adult ICU patients (≥18 years) with continuous RR monitoring and an ICU stay >24 h. Exclusion criteria were a non-first ICU admission (MIMIC-IV and eICU) and unavailable RR data. Because the cohort was restricted to patients with an ICU stay longer than 24 h, all included patients survived to the 24-hour landmark and none was excluded on that basis. For HiRID, patients with any RR monitoring gap exceeding 6 h were additionally excluded, and for retained patients all RR data following the first gap exceeding 2 h were removed, to preserve continuous monitoring periods.

### Data processing

Missing data were imputed using median values (Supplementary Table S1); multiple imputation by chained equations was performed as a sensitivity analysis. Laboratory covariates comprised haemoglobin, white blood cell count, platelet count, lactate and creatinine, all taken as the extreme value recorded within the first 24 h after ICU admission rather than over the entire ICU stay, so that covariates could not be affected by exposure accumulated later in the stay. RR values were winsorised across all databases: values <5 breaths/min were set to 5 and values >40 breaths/min were set to 40. These thresholds were chosen to limit the influence of monitoring artefacts: values below 5 breaths/min in continuously monitored patients most often reflect apnoea alarms or sensor detachment rather than a sustained physiological rate, and values above 40 breaths/min are similarly dominated by signal artefact. Winsorisation affected 0.39% and 0.40% of MIMIC-IV records at the lower and upper bounds respectively. For HiRID, a two-step gap rule was applied as described above.

### RR exposure intensity-duration analysis (heatmap)

A visualisation method was developed to examine RR exposure intensity and duration in relation to mortality. Because a continuous episode of a given duration can only be observed if monitoring continues for at least that long, this analysis used exposure accumulated over the entire ICU monitoring period rather than the 24-hour landmark window used elsewhere; the same computation restricted to the first 24 h is reported as a sensitivity analysis (Supplementary Figure S4). RR ranges were defined as 15 consecutive, non-overlapping 2-breaths/min intervals from 5 to 35 breaths/min, with durations from 0 to 120 min at 3-minute intervals. For each patient, exposure events were counted for each RR range-duration combination, where an event was defined as continuous RR within that range for at least the specified duration. Intervals between consecutive recordings exceeding 5 min contributed no exposure time and interrupted continuous episodes, so that monitoring gaps could not inflate estimated exposure duration. Total events for survivors and non-survivors were recorded separately. For each cell we computed the ratio of the mean number of events per non-survivor to the mean number per survivor; this quantity is an event-rate ratio rather than a conventional odds ratio, and is used here descriptively. Cells containing fewer than 5 exposure events in total were treated as missing, and the surface was smoothed with a Gaussian filter (*σ* = 0.5) for contour rendering only. Deviation values were calculated by subtracting the mean ratio across all valid cells from each cell. Because a single sustained episode is counted at every duration threshold it exceeds, counts in adjacent duration columns are nested and not independent; the heatmap is therefore presented as an exploratory, hypothesis-generating analysis, with formal inference based on the regression models below. Per-cell event counts are reported in Supplementary Figure S3 and Supplementary Table S7.

### RR range-specific exposure duration analysis

The percentage of monitoring time spent in each of eight RR ranges (<10, 10–12, 12–20, 20–24, 24–27, 27–30, 30–35, >35 breaths/min) was calculated as (time in range/total monitoring time) × 100. Because respiratory rate in MIMIC-IV and eICU is recorded as intermittent observations rather than continuously, each recording was assumed to persist until the next recording, and the final recording of each patient was assigned a fixed duration of 60 min; total monitoring time was the sum of these durations. No interpolation of values between recordings was performed. In HiRID, which provides near-continuous monitoring, the same rule was applied together with the gap handling described above. The boundaries follow established early-warning-score categories, in which 12–20 breaths/min constitutes the reference band scored as normal, and were further subdivided above 20 breaths/min to resolve the graded risk observed in that region; the threshold of 27 breaths/min corresponds to the cut-off previously shown to predict in-hospital cardiopulmonary arrest. Because the eight proportions form a closed composition summing to 100%, a model containing all eight simultaneously is not identifiable. Each range was therefore modelled separately, so that its coefficient reflects the effect of increasing time in that range with the remaining time redistributed as observed. A reference-category model omitting the 12–20 breaths/min range, in which the coefficient reflects reallocating one percentage point of monitoring time from the optimal range to the range of interest, is reported as a sensitivity analysis (Supplementary Table S8). Multivariable Cox models examined associations with 28-day mortality in MIMIC-IV using stepwise adjustment. Analyses in eICU used logistic regression with in-hospital mortality as the outcome.

### Restricted cubic spline analysis

Restricted cubic spline (RCS) analysis examined the continuous association between the time-weighted mean RR and mortality in MIMIC-IV. The time-weighted mean was calculated as *Σ*(RR × duration)/*Σ*(duration) across all recordings within the exposure window, where the duration attributed to each recording was the interval to the next recording. Splines were fitted with five knots placed at the 5th, 27.5th, 50th, 72.5th and 95th percentiles of the exposure distribution, and the reference value was set at the nadir of the fitted curve rather than at an arbitrary pre-specified value. Multivariable Cox models were adjusted as in the fully adjusted model above. Departure from linearity was assessed by a likelihood ratio test comparing the spline model with a model containing only the linear term.

### Statistical analysis

Continuous variables are presented as median (IQR) and compared using the Wilcoxon rank-sum test. Categorical variables are presented as numbers (percentages) and compared using the chi-square test. Analyses were performed using Python 3.13 with NumPy, Pandas, Matplotlib, lifelines and statsmodels. Cox models were fitted by maximum partial likelihood without penalisation, with covariates standardised to ensure numerical stability. Discrimination of the logistic models was evaluated using the area under the receiver operating characteristic curve. The proportional hazards assumption was assessed using scaled Schoenfeld residuals, and period-specific hazard ratios are reported where the assumption was not met. Correction for multiple comparisons across the 8 ranges and 5 models was performed using both Bonferroni and Benjamini-Hochberg procedures. In eICU, robust standard errors accounting for clustering of patients within hospitals were computed, and a Fine-Gray subdistribution hazards model treating discharge alive as a competing event was fitted as a sensitivity analysis. Invasive mechanical ventilation in eICU was ascertained within the exposure window from care-plan entries, treatment entries and recorded artificial airways, with non-invasive ventilation excluded (Supplementary Table S11). All tests were two-sided with *P* < 0.05 considered significant. Hazard ratios and odds ratios are reported per 1 percentage-point increase in exposure time.

## Results

### Study population and baseline characteristics

A total of 160,075 critically ill patients were included across three cohorts: the HiRID cohort (11,717 patients) for the intensity-duration heatmap analysis (Figure 1), the MIMIC-IV cohort (53,886 patients) for the primary landmark analysis and restricted cubic spline analysis (Supplementary Figure S1), and the eICU cohort (94,472 patients) for analysis in an independent population (Supplementary Figure S2). Because all cohorts required an ICU stay longer than 24 h, every patient contributed a complete 24-hour exposure window and none was excluded at the landmark. Ten patients whose laboratory values were recorded as non-positive could not enter the adjusted models, so all regression and spline analyses in MIMIC-IV were fitted in 53,876 patients (6,796 deaths).

**Figure 1:**
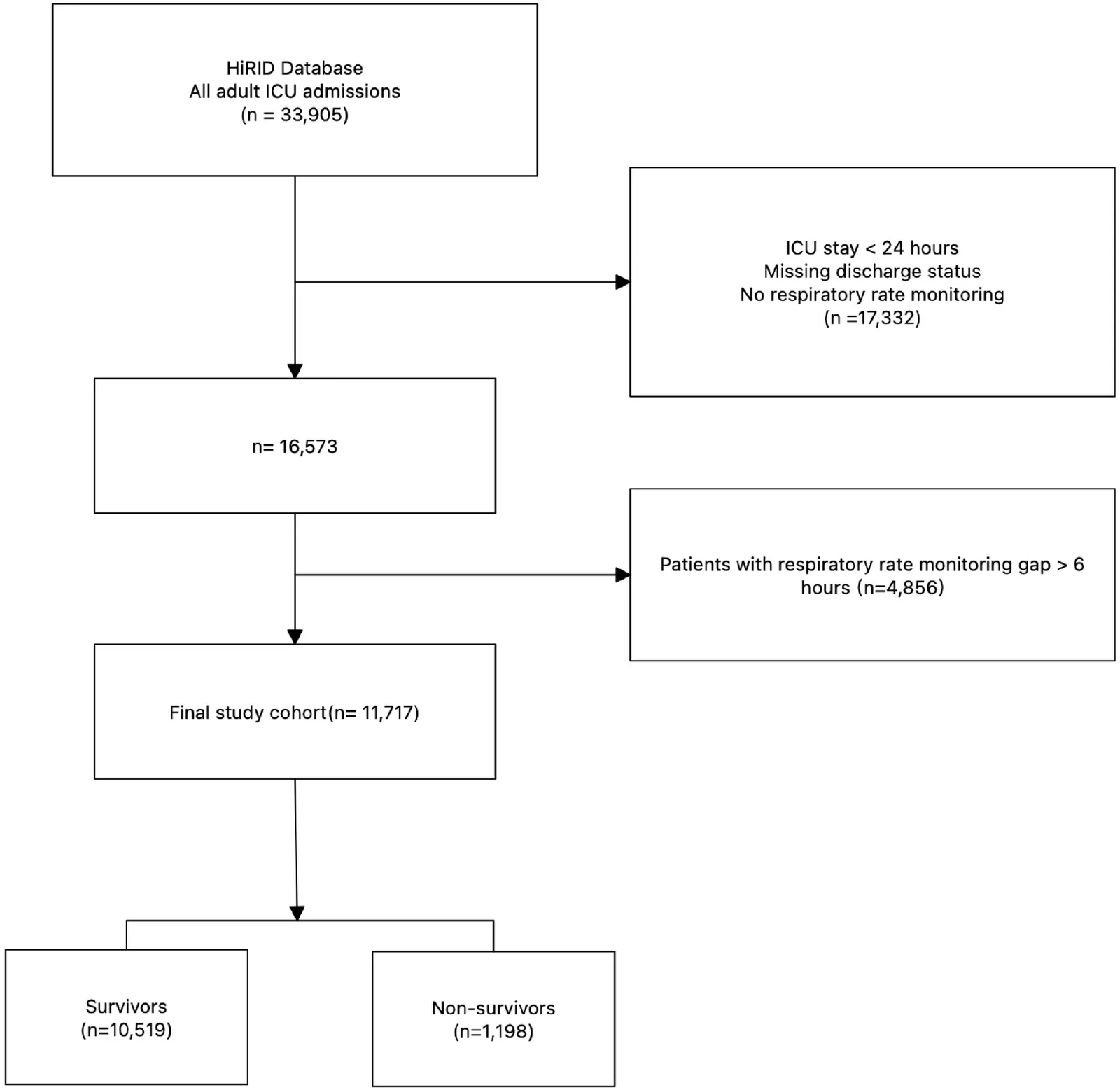
Patient selection flowchart for the HiRID cohort.

In the HiRID cohort, 1,198 patients (10.2%) died in hospital. Non-survivors had significantly higher SOFA scores, higher maximum lactate levels, higher maximum white blood cell counts, lower minimum platelet counts, and higher maximum creatinine levels compared with survivors (Table 1). In the MIMIC-IV database, 6,797 patients (12.6%) died within 28 days; in the eICU database, 8,820 patients (9.3%) died during hospitalization. Across all cohorts, non-survivors consistently exhibited higher SOFA scores and more deranged laboratory values (Supplementary Table S2).

**Table 1:** Baseline characteristics of the study population stratified by survival status in HiRID database.

Characteristic	Survivors (*n* = 10,519)	Non-survivors (*n* = 1,198)	*P* value
Demographics			
Age, years	65.00 (55.00, 75.00)	70.00 (55.00, 75.00)	0.069
Male sex, *n* (%)	6,856 (65.2)	752 (62.8)	0.105
Severity			
SOFA score	6.00 (5.00, 8.00)	9.00 (7.00, 11.00)	<0.001
Laboratory variables			
Minimum hemoglobin, g/dL	9.20 (8.10, 10.60)	9.10 (7.80, 11.00)	0.042
Maximum WBC, 10^9^/L	12.10 (9.20, 15.40)	14.00 (10.70, 19.50)	<0.001
Minimum platelet, 10^9^/L	147.00 (102.00, 205.00)	129.00 (69.00, 177.00)	<0.001
Maximum lactate, mmol/L	2.20 (1.40, 3.20)	4.20 (2.50, 7.80)	<0.001
Maximum creatinine, mg/dL	0.97 (0.74, 1.39)	1.26 (0.85, 2.30)	<0.001

Values are presented as median (interquartile range) for continuous variables and number (percentage) for categorical variables. *P* values were calculated using the Wilcoxon rank-sum test for continuous variables and the chi-square test for categorical variables. Laboratory values are the extreme recorded over the ICU stay, with missing values imputed by the median (Supplementary Table S1); HiRID contributed only the descriptive intensity-duration analysis, in which no covariate adjustment was performed. SOFA: Sequential Organ Failure Assessment; WBC: white blood cell count.

### Heatmap analysis

We developed a heatmap approach to visualise the relationship between RR exposure patterns and mortality (Figure 2). Because a continuous episode of a given duration can only be observed if monitoring continues for at least that long, this analysis retained exposure accumulated over the entire ICU monitoring period rather than the 24-hour landmark window; the corresponding landmark analysis is shown in Supplementary Figure S4. Of the 11,717 patients in the HiRID cohort, 10,696 (1,172 non-survivors) contributed at least one exposure event and are represented in the grid. The heatmap (Figure 3) revealed three distinct zones: the 12–20 breaths/min zone displayed deep blue colouring (deviation −4.3 to −1.5), indicating a lower relative event rate among non-survivors; the tachypnoeic zone (RR >27 breaths/min) showed a progressive transition from orange to deep red with prolonged exposure; and the bradypnoeic zone (RR <9 breaths/min) showed a time-dependent pattern, with deviation increasing from −3.2 to −1.7 at brief exposures to positive values with sustained exposure. Per-cell event counts varied by more than four orders of magnitude (median 284 events per cell, range 15–1,420,714). Cells with fewer than 20 exposure events are hatched in Figure 3 and enumerated in Supplementary Figure S3; all 12 such cells lie in the 5–7 breaths/min band at durations of 87 min or longer, and the corresponding estimates rest on 15–18 events contributed by only 13 patients (7 non-survivors). The sustained-bradypnoea region of the heatmap should therefore be regarded as exploratory. Brief exposures to low RR were dominated by artefact: 7.8% of HiRID recordings were below 5 breaths/min and 6.7% were exactly zero, and after winsorisation these accounted for 95.7% of the events in the 5–7 breaths/min band at the shortest duration threshold, whereas at 30 min and beyond the corresponding events were almost entirely genuine.

**Figure 2:**
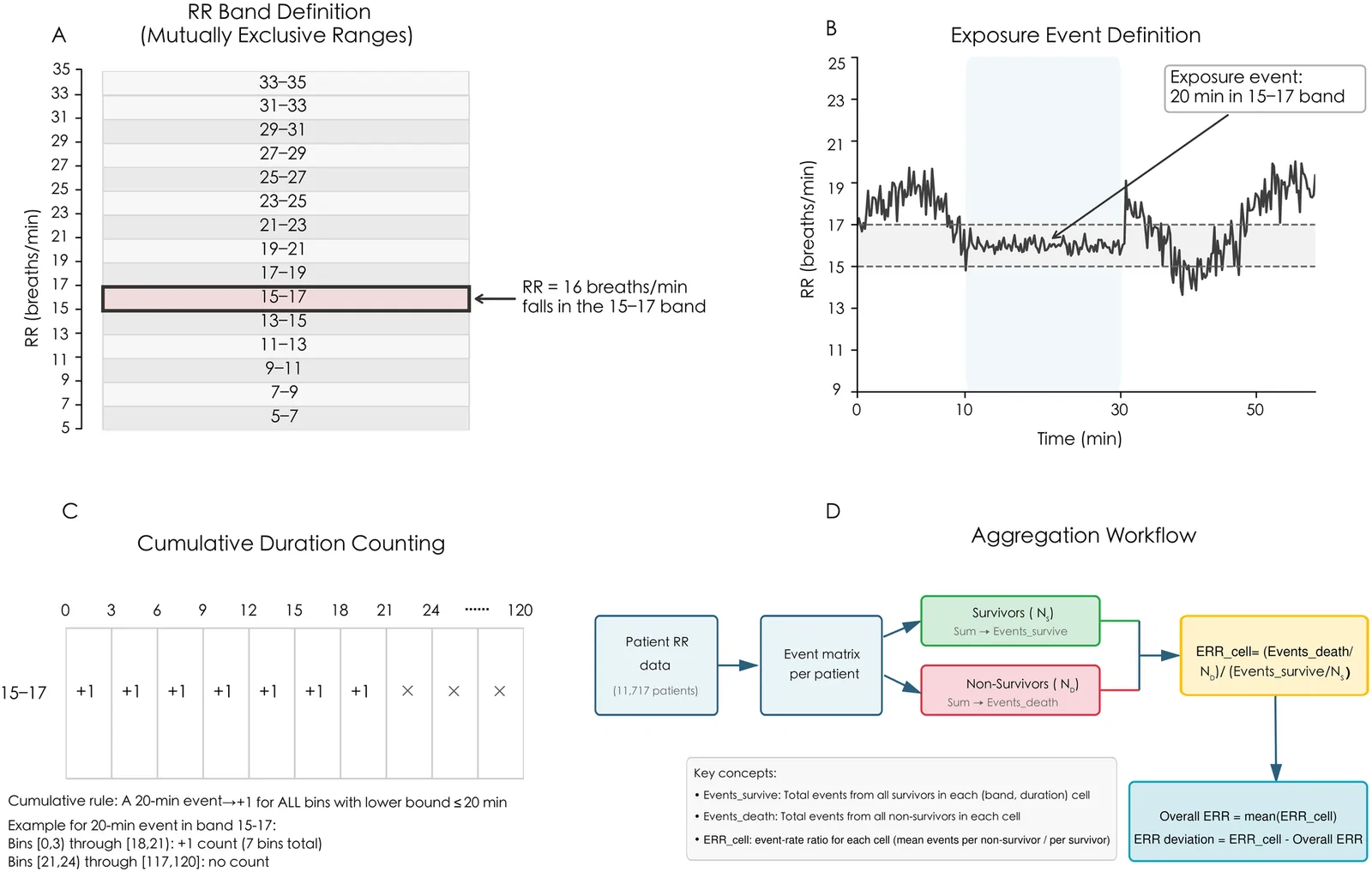
Schematic overview of RR exposure heatmap construction methodology. **(A)** Shows 15 mutually exclusive RR bands (5–7 to 33–35 breaths/min). **(B)** Demonstrates the exposure event definition, in which continuous RR maintenance within a specific band constitutes an exposure event. **(C)** Illustrates the cumulative counting logic across duration thresholds. **(D)** Outlines the workflow for calculating the event-rate ratio and its deviation from the overall mean.

**Figure 3:**
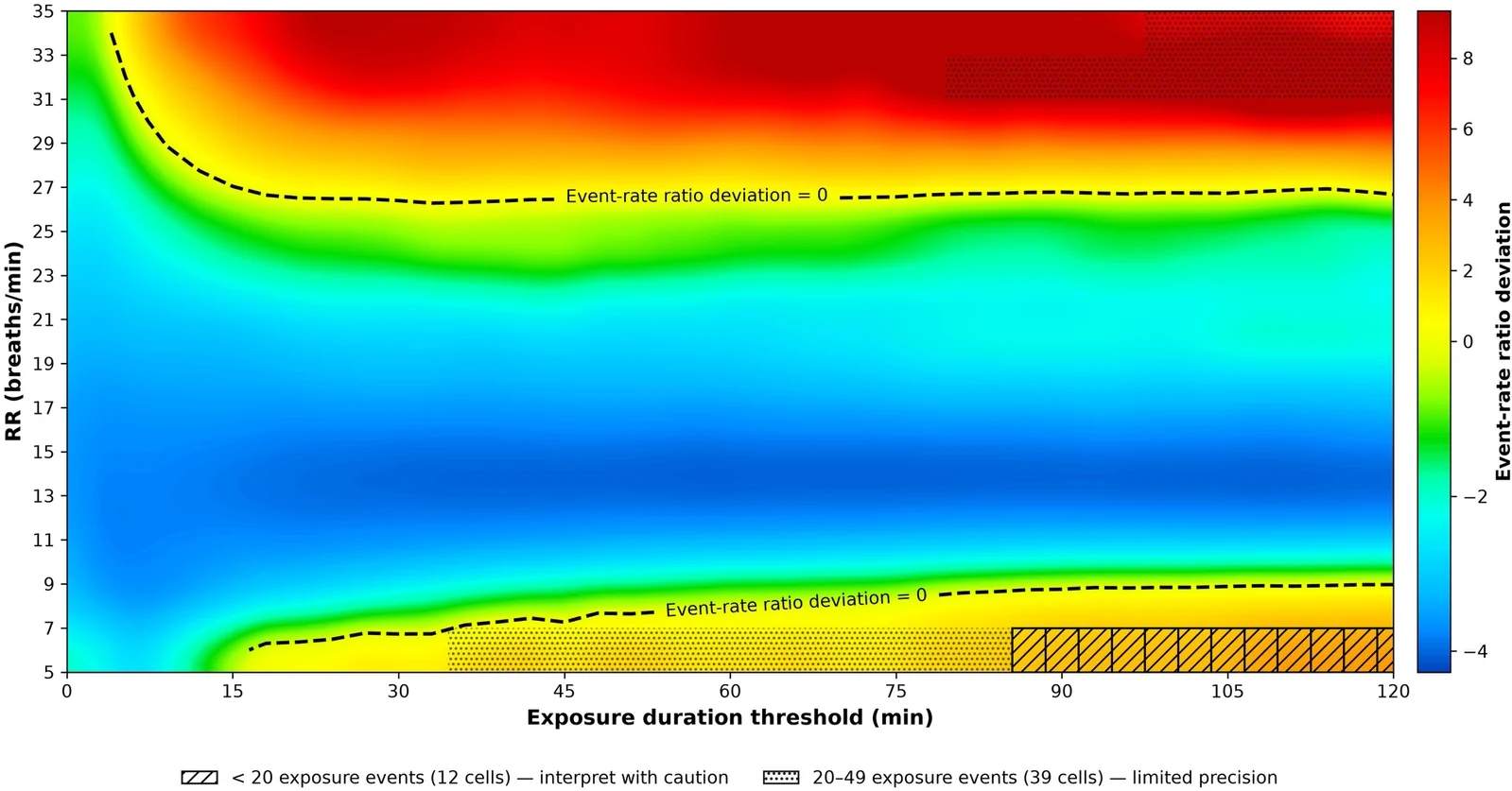
RR exposure intensity-duration heatmap [HiRID; 11,717-patient cohort, of whom 10,696 patients (1,172 non-survivors) contributed at least one exposure event]. Exposure was accumulated over the entire ICU monitoring period; the corresponding 24-hour landmark analysis is shown in Supplementary Figure S4. Colour represents the deviation of each cell's event-rate ratio from the overall mean across all valid cells; blue indicates a lower and red a higher relative event rate among non-survivors, and dashed lines mark a deviation of zero. Cells were retained only if they contained at least 5 exposure events, and the surface was smoothed with a Gaussian filter (*σ* = 0.5) for contour rendering. Hatching marks cells with limited statistical support: diagonal hatching, fewer than 20 exposure events (12 cells); dotted hatching, 20–49 events (39 cells). Per-cell event counts are given in Supplementary Figure S3 and Supplementary Table S7. The apparently elevated event rate in the sustained bradypnoea region (5–7 breaths/min for 87 min or longer) rests on 15–18 exposure events and should be regarded as exploratory.

### Cox regression analysis

Cox proportional hazards analysis evaluated the associations between the percentage of the first 24 h spent in different RR ranges and subsequent 28-day mortality in the MIMIC-IV cohort (Table 2, Figure 4). Five progressive models were constructed to assess the robustness of associations. After comprehensive adjustment for demographics, disease severity, laboratory findings and comorbidities (Model 5), a greater proportion of time in the 12–20 bpm range was associated with lower mortality (HR 0.988, 95% CI 0.987–0.989, *P* < 0.001), corresponding to a 1.2% lower hazard for each 1 percentage-point increase in time spent in this range. Time in the 10–12 bpm range showed a similar association (HR 0.981, 95% CI 0.977–0.986, *P* < 0.001).

**Table 2:** Association between the percentage of the first 24 h spent in each RR range and subsequent 28-day mortality: Cox models under the landmark design (MIMIC-IV).

RR Range	Model 1		Model 2		Model 3		Model 4		Model 5	
	HR (95% CI)	*P*	HR (95% CI)	*P*	HR (95% CI)	*P*	HR (95% CI)	*P*	HR (95% CI)	*P*
<10 bpm	0.989 (0.983–0.994)	<0.001	0.995 (0.990–1.001)	0.096	0.987 (0.981–0.992)	<0.001	0.988 (0.982–0.993)	<0.001	0.990 (0.984–0.996)	<0.001
10–12 bpm	0.972 (0.967–0.976)	<0.001	0.977 (0.973–0.982)	<0.001	0.977 (0.972–0.981)	<0.001	0.979 (0.975–0.984)	<0.001	0.981 (0.977–0.986)	<0.001
12–20 bpm	0.984 (0.983–0.984)	<0.001	0.984 (0.983–0.984)	<0.001	0.985 (0.984–0.986)	<0.001	0.986 (0.985–0.987)	<0.001	0.988 (0.987–0.989)	<0.001
20–24 bpm	1.006 (1.005–1.008)	<0.001	1.005 (1.003–1.006)	<0.001	1.004 (1.003–1.006)	<0.001	1.004 (1.003–1.005)	<0.001	1.003 (1.002–1.005)	<0.001
24–27 bpm	1.019 (1.018–1.021)	<0.001	1.020 (1.018–1.021)	<0.001	1.017 (1.016–1.018)	<0.001	1.015 (1.013–1.016)	<0.001	1.012 (1.011–1.014)	<0.001
27–30 bpm	1.028 (1.026–1.030)	<0.001	1.029 (1.027–1.031)	<0.001	1.026 (1.024–1.028)	<0.001	1.022 (1.020–1.024)	<0.001	1.019 (1.017–1.021)	<0.001
30–35 bpm	1.027 (1.025–1.028)	<0.001	1.030 (1.028–1.032)	<0.001	1.027 (1.025–1.028)	<0.001	1.022 (1.020–1.024)	<0.001	1.019 (1.017–1.021)	<0.001
>35 bpm	1.027 (1.025–1.030)	<0.001	1.033 (1.030–1.035)	<0.001	1.031 (1.029–1.034)	<0.001	1.029 (1.026–1.031)	<0.001	1.026 (1.023–1.028)	<0.001

Exposure was measured during the first 24 h after ICU admission and follow-up began at the 24-hour landmark. *n* = 53,876; 6,796 deaths. Model 1: unadjusted; Model 2: adjusted for age and sex; Model 3: Model 2 plus SOFA score; Model 4: Model 3 plus laboratory values (haemoglobin, WBC, platelet count, lactate, creatinine, all measured within the first 24 h); Model 5: Model 4 plus comorbidities (diabetes, hypertension, renal dysfunction, COPD). Models were fitted by maximum partial likelihood without penalisation. HR, hazard ratio; CI, confidence interval; SOFA, sequential organ failure assessment.

**Figure 4:**
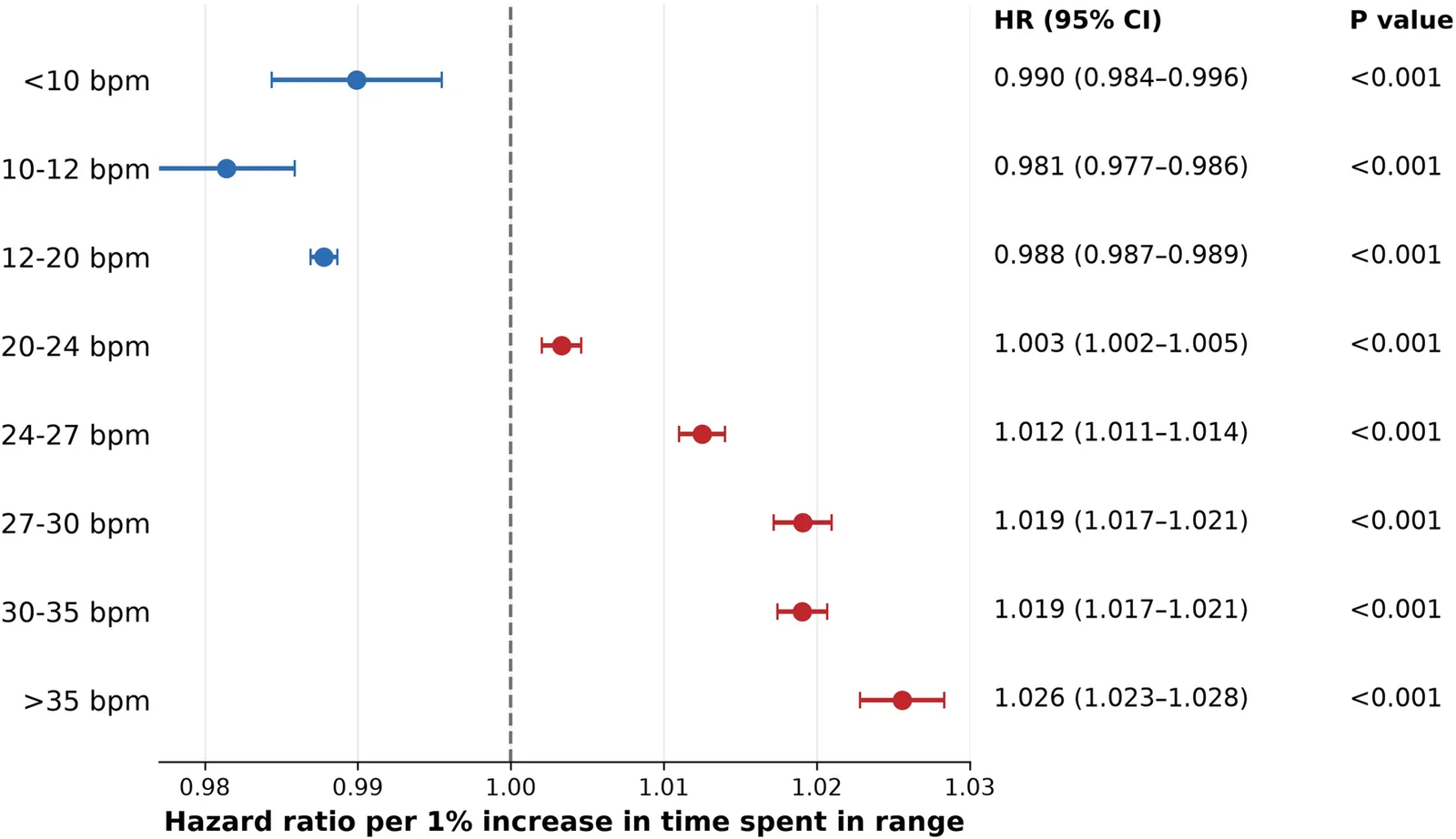
Forest plot displaying hazard ratios (95% CI) for 28-day mortality associated with the percentage of the first 24 h of monitoring time spent in different RR ranges, under the landmark design. Estimates are from the fully adjusted model. The vertical dashed line indicates a hazard ratio of 1.0.

Risk increased progressively across the tachypnoeic ranges: 20–24 bpm (HR 1.003, 95% CI 1.002–1.005), 24–27 bpm (HR 1.012, 95% CI 1.011–1.014), 27–30 bpm (HR 1.019, 95% CI 1.017–1.021), 30–35 bpm (HR 1.019, 95% CI 1.017–1.021) and >35 bpm (HR 1.026, 95% CI 1.023–1.028; all *P* < 0.001). Time spent below 10 breaths/min was associated with slightly lower rather than higher mortality (HR 0.990, 95% CI 0.984–0.996). This apparent benefit did not persist once the comparison was made explicit: when the range proportions were entered simultaneously with 12–20 breaths/min omitted as the reference, so that each coefficient reflects reallocating one percentage point of monitoring time away from the optimal range, the direction reversed for the low ranges (<10 breaths/min HR 1.006, 95% CI 1.001–1.012) while the tachypnoeic associations were essentially unchanged (Supplementary Table S8). These associations retained their direction and gradient after further adjustment for the Charlson comorbidity index, congestive heart failure, liver disease, malignancy, immunosuppression, Sepsis-3, do-not-resuscitate status, vasoactive agents, acute respiratory distress syndrome and pneumonia (Supplementary Table S4). Effect sizes for the 12–20 breaths/min range and for all tachypnoeic ranges attenuated by 13%–23% while directions, gradients and statistical significance were preserved; the two lowest ranges changed less (10–12 breaths/min, 8.7% attenuation) or marginally in the opposite direction (<10 breaths/min, 2.2%).

### Analysis in an independent cohort

Analysis of the eICU cohort, in which the same exposure definition was applied but the outcome was in-hospital rather than 28-day mortality and logistic rather than Cox regression was used, showed associations of the same direction and gradient (Table 3). A greater proportion of the first 24 h spent in the 12–20 bpm range was associated with lower in-hospital mortality (OR 0.991, 95% CI 0.990–0.991), as was time in the 10–12 bpm range (OR 0.983, 95% CI 0.980–0.987). Because the outcome definitions and modelling approaches differ between databases, these analyses are presented as an assessment of transferability across outcomes and populations rather than as external validation of a fixed prediction model.

**Table 3:** Association between the percentage of the first 24 h spent in each RR range and in-hospital mortality (eICU).

Time percentage in RR range	Univariate analysis				Multivariable analysis			
	OR	95% CI	*P*	AUC	OR	95% CI	Cluster-robust 95% CI (P)	AUC
RR <10 bpm	1.001	(0.997–1.005)	0.573	0.499	0.995	(0.991–0.999)	(0.990–1.000) 0.053	0.788
RR 10–12 bpm	0.977	(0.973–0.981)	<0.001	0.543	0.983	(0.980–0.987)	(0.978–0.988) <0.001	0.789
RR 12–20 bpm	0.986	(0.986–0.987)	<0.001	0.620	0.991	(0.990–0.991)	(0.989–0.992) <0.001	0.796
RR 20–24 bpm	1.000	(0.999–1.001)	0.886	0.494	1.000	(0.999–1.001)	(0.998–1.001) 0.787	0.788
RR 24–27 bpm	1.016	(1.015–1.018)	<0.001	0.590	1.011	(1.010–1.013)	(1.010–1.013) <0.001	0.792
RR 27–30 bpm	1.023	(1.021–1.024)	<0.001	0.598	1.017	(1.015–1.019)	(1.014–1.020) <0.001	0.793
RR 30–35 bpm	1.027	(1.026–1.029)	<0.001	0.601	1.019	(1.017–1.021)	(1.016–1.022) <0.001	0.795
RR >35 bpm	1.023	(1.021–1.025)	<0.001	0.563	1.020	(1.017–1.022)	(1.016–1.023) <0.001	0.792

Exposure was defined as in Table 2. *n* = 94,472; 8,820 in-hospital deaths across 201 hospitals. Multivariable models were adjusted for age, sex, SOFA score, laboratory values measured within the first 24 h, and comorbidities (diabetes, hypertension, renal dysfunction, COPD). Cluster-robust confidence intervals and the accompanying *P* values account for clustering of patients within the 201 contributing hospitals. OR, odds ratio; CI, confidence interval; AUC, area under the ROC curve; SOFA, sequential organ failure assessment.

Risk again increased progressively across the tachypnoeic ranges: 24–27 bpm (OR 1.011, 95% CI 1.010–1.013), 27–30 bpm (OR 1.017, 95% CI 1.015–1.019), 30–35 bpm (OR 1.019, 95% CI 1.017–1.021) and >35 bpm (OR 1.020, 95% CI 1.017–1.022; all *P* < 0.001), whereas time below 10 breaths/min was not associated with increased mortality (OR 0.995, 95% CI 0.991–0.999). Accounting for clustering of patients within the 201 contributing hospitals widened confidence intervals by a factor of 1.16–1.75. The associations for the 10–12 and 12–20 breaths/min ranges and for all tachypnoeic ranges remained statistically significant, whereas the association for time below 10 breaths/min did not (cluster-robust *P* = 0.053) and time in the 20–24 breaths/min range remained unassociated with mortality (cluster-robust *P* = 0.787). A Fine-Gray subdistribution hazards model treating discharge alive as a competing event yielded consistent estimates (Supplementary Table S9). Under the landmark design a model containing only conventional predictors reached an area under the curve of 0.788, indicating that most of the overall discrimination was attributable to these conventional variables rather than to respiratory-rate exposure. Adding the RR exposure variables raised it to 0.801 (ΔAUC 0.013; likelihood ratio *χ*^2^ = 934.7, 7 df, *P* < 0.001), with an integrated discrimination improvement of 0.014, a continuous net reclassification improvement of 0.33, and a fall in the Brier score from 0.0726 to 0.0715. Because the continuous net reclassification improvement is known to be biased upward, we base this interpretation on the change in the area under the curve and the likelihood ratio test, and regard the gain as modest but consistent.

### Dose-response and sensitivity analyses

Restricted cubic spline analysis of the time-weighted mean RR during the first 24 h revealed a significantly non-linear, markedly asymmetric relationship with 28-day mortality (*P* for non-linearity <0.001; Figure 5). Risk was lowest at 16.3 breaths/min, and the range statistically indistinguishable from this nadir extended from 13.9 to 17.4 breaths/min. Above the nadir the adjusted hazard ratio rose steeply, reaching 1.38 at 20 breaths/min, 2.19 at 24 breaths/min and 3.67 at 30 breaths/min. Below the nadir the curve was much flatter, with a hazard ratio of only 1.18 at 12.4 breaths/min. The corresponding curve obtained when exposure was accumulated over the entire ICU stay is shown in Supplementary Figure S5.

**Figure 5:**
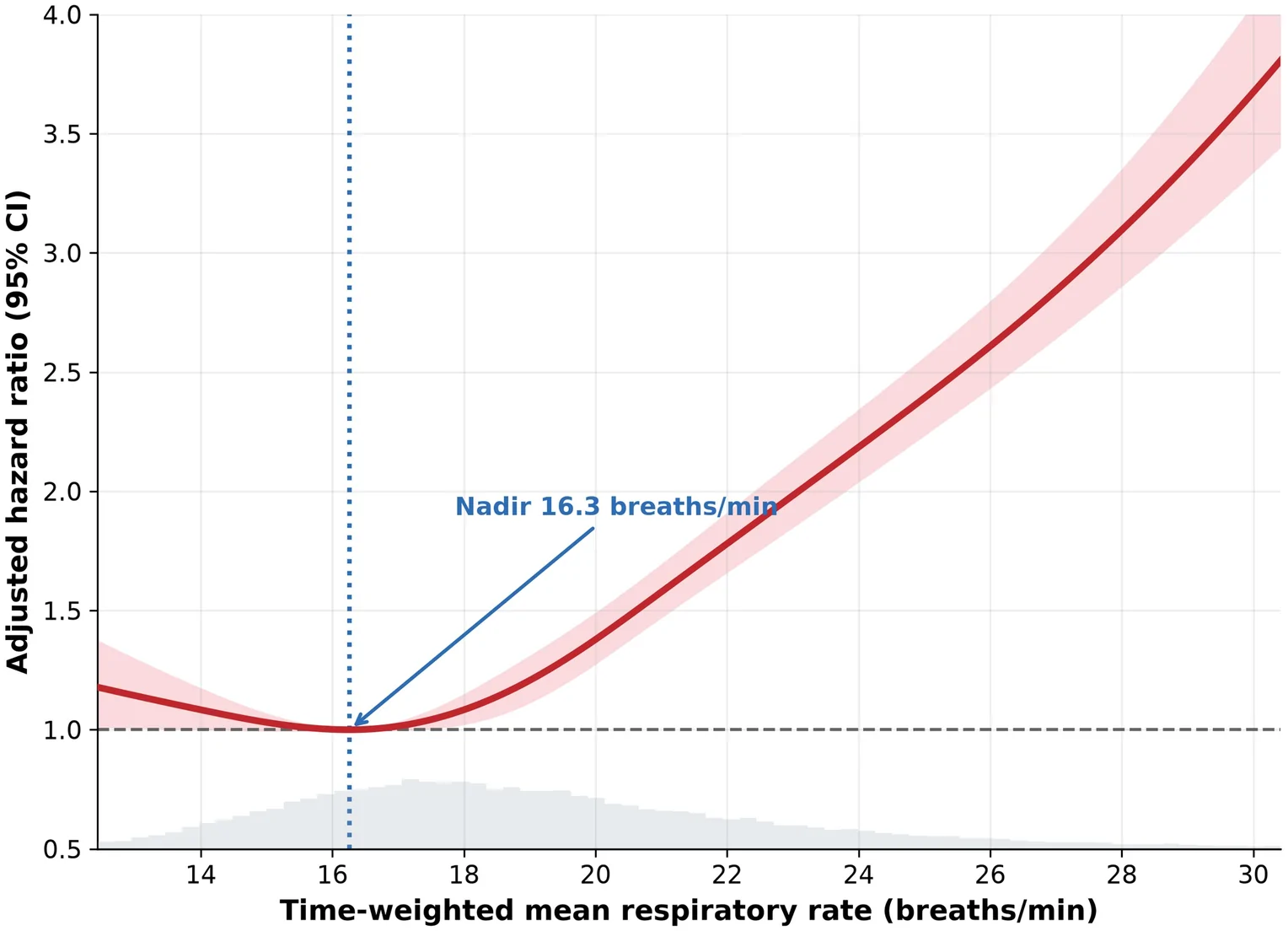
Restricted cubic spline curve of the adjusted hazard ratio for 28-day mortality across the time-weighted mean respiratory rate during the first 24 h (MIMIC-IV). Splines were fitted with five knots at the 5th, 27.5th, 50th, 72.5th and 95th percentiles, and the reference value was set at the nadir of the fitted curve (16.3 breaths/min). Models were fitted in 53,876 patients (6,796 deaths) and adjusted for age, sex, SOFA score, laboratory values measured within the first 24 h, and comorbidities. The solid line denotes the adjusted hazard ratio and the shaded band its 95% confidence interval; the horizontal dashed line marks a hazard ratio of 1.0 and the vertical dotted line the nadir. The grey histogram shows the distribution of the exposure. The association was significantly non-linear (*P* < 0.001) and markedly asymmetric.

Sensitivity analyses addressed the temporal ordering of exposure and outcome (Table 4). When exposure was instead accumulated over the entire ICU stay, a greater proportion of time below 10 breaths/min appeared to be associated with higher mortality (HR 1.029, 95% CI 1.024–1.035). This association attenuated monotonically as respiratory-rate data from the final 6, 12 and 24 h before death or discharge were progressively excluded, and was absent under the landmark design (HR 0.990); the same pattern was observed in eICU, and the corresponding heatmap analyses in HiRID are shown in Supplementary Figure S4. Associations for the tachypnoeic ranges attenuated by approximately half but retained their direction, gradient and statistical significance across all exposure definitions. Results were unchanged when stratified by mechanical ventilation and by sedative or opioid exposure in MIMIC-IV (Supplementary Table S3 and Supplementary Figure S8), and after multiple imputation of missing laboratory values (Supplementary Table S5). Stratification by mechanical ventilation was also possible in eICU, where invasive ventilation during the exposure window was ascertained for every patient by combining care-plan, treatment and artificial-airway records, and was present in 31.3% of the cohort, a prevalence comparable with that in MIMIC-IV (29.2%); the tachypnoeic associations were present in both strata with the same direction and gradient (Supplementary Table S11 and Supplementary Figure S8). The proportional hazards assumption was not met for three of the eight ranges; all eight showed the same pattern of attenuation towards the null over follow-up, and period-specific estimates are reported in Supplementary Table S5 and Supplementary Figure S7. All associations remained significant after Bonferroni and false-discovery-rate correction across the 40 tests performed. To address the differing outcome definitions between databases directly, we also repeated the MIMIC-IV analysis using in-hospital mortality and logistic regression, matching the specification used in eICU (5,277 in-hospital deaths among 53,876 patients). The associations were unchanged in direction and gradient (12–20 breaths/min OR 0.985, 95% CI 0.984–0.986; >35 breaths/min OR 1.034, 95% CI 1.030–1.038; Supplementary Table S10).

**Table 4:** Sensitivity of the exposure-mortality associations to the definition of the exposure window.

RR range	MIMIC-IV (HR, 95% CI)			eICU (OR, 95% CI)		
	Full ICU stay	Excluding last 24 h	24-h landmark	Full ICU stay	Excluding last 24 h	24-h landmark
<10 bpm	1.029 (1.024–1.034)	1.003 (0.996–1.009)	0.990 (0.984–0.996)	1.015 (1.011–1.019)	0.999 (0.994–1.004)	0.995 (0.991–0.999)
10–12 bpm	0.986 (0.981–0.991)	0.977 (0.972–0.983)	0.981 (0.977–0.986)	0.983 (0.979–0.988)	0.980 (0.975–0.985)	0.983 (0.980–0.987)
12–20 bpm	0.981 (0.980–0.982)	0.984 (0.983–0.985)	0.988 (0.987–0.989)	0.986 (0.985–0.987)	0.988 (0.987–0.989)	0.991 (0.990–0.991)
20–24 bpm	0.997 (0.996–0.999)	1.002 (1.000–1.003)	1.003 (1.002–1.005)	0.995 (0.993–0.996)	0.998 (0.996–0.999)	1.000 (0.999–1.001)
24–27 bpm	1.027 (1.025–1.029)	1.022 (1.020–1.024)	1.012 (1.011–1.014)	1.018 (1.016–1.019)	1.017 (1.015–1.019)	1.011 (1.010–1.013)
27–30 bpm	1.040 (1.038–1.043)	1.032 (1.030–1.035)	1.019 (1.017–1.021)	1.026 (1.024–1.029)	1.024 (1.021–1.026)	1.017 (1.015–1.019)
30–35 bpm	1.042 (1.041–1.044)	1.034 (1.032–1.036)	1.019 (1.017–1.021)	1.033 (1.031–1.035)	1.029 (1.026–1.031)	1.019 (1.017–1.021)
>35 bpm	1.047 (1.043–1.050)	1.035 (1.031–1.038)	1.026 (1.023–1.028)	1.034 (1.032–1.037)	1.027 (1.024–1.029)	1.020 (1.017–1.022)

MIMIC-IV values are hazard ratios for 28-day mortality (*n* = 53,876; 6,796 deaths); eICU values are odds ratios for in-hospital mortality (*n* = 94,472; 8,820 deaths). Covariates were ascertained within the first 24 h in every column, so the landmark columns correspond exactly to Tables 2, 3. The exclusion columns retain only patients with at least 12 h of monitoring after truncation (MIMIC-IV *n* = 39,637 with 5,994 deaths; eICU *n* = 66,908 with 7,175 deaths).

## Discussion

In this analysis of 160,075 ICU patients across three international databases, a greater cumulative burden of tachypnoea during the first 24 h of ICU admission was associated with higher subsequent mortality, and a greater proportion of time spent between 12 and 20 breaths/min was associated with lower mortality. These findings extend previous observations that respiratory rate serves as a prognostic indicator in critically ill patients. While earlier studies established the value of single-point RR measurements in predicting adverse outcomes (18, 19), our work provides large-scale, multi-database evidence that the duration as well as the intensity of RR deviations carries prognostic information, and that this information is not merely a reflection of the dying process: the associations for the tachypnoeic ranges persisted under a landmark design in which exposure and outcome windows do not overlap, and after adjustment for mechanical ventilation, sedation, comorbidity burden, sepsis and treatment limitations.

The strength of association between sustained RR exposure and mortality exhibited clear time-dependent patterns. Our heatmap analysis suggested that brief episodes of extreme RR values were accompanied by little excess mortality, whereas sustained exposure was accompanied by progressively higher mortality. This temporal pattern was apparent at both ends of the RR spectrum. However, the interpretation of the two ends differs substantially. For tachypnoea the pattern was supported by large numbers of exposure events and persisted under every sensitivity analysis we performed. For bradypnoea the corresponding cells rested on 15–18 events, brief low-RR exposures were dominated by winsorised artefact, and the association disappeared once exposure was restricted to the first 24 h. The bradypnoeic findings should therefore be regarded as exploratory and hypothesis-generating.

Early warning scores such as the National Early Warning Score (NEWS) show good discrimination for unanticipated ICU admission and short-term mortality (5); however, these systems assess respiratory rate categorically at single time points. NEWS appropriately recognizes both tachypnea and bradypnea as risk indicators, but our findings suggest that the duration of RR abnormalities carries prognostic information beyond threshold-based alerts. The association between sustained exposure to RR >27 breaths/min and mortality extends existing frameworks by quantifying the cumulative burden of tachypnoea over time rather than relying solely on instantaneous values. We make no corresponding claim for low respiratory rates, because that association did not survive the landmark design.

The relationship between RR and mortality was markedly asymmetric. Above the spline nadir of 16.3 breaths/min the adjusted hazard ratio rose steeply, whereas below it the curve was comparatively flat. This differential likely reflects distinct underlying pathophysiological mechanisms. Prolonged tachypnoea may indicate worsening respiratory mechanics, increased dead space ventilation, or progressive respiratory muscle fatigue that can precipitate respiratory failure (20, 21). Sustained bradypnoea in ICU patients is much less common and, when observed, is frequently iatrogenic, reflecting oversedation or controlled ventilation rather than primary respiratory pathology. Consistent with this interpretation, the association between low RR and mortality was confined to analyses in which exposure could be measured shortly before death, and the interval whose association differed most between ventilated and spontaneously breathing patients was 10–12 breaths/min (hazard ratio 0.967 vs. 0.989), in which a low rate in ventilated patients is more likely to represent a set ventilator frequency than suppressed respiratory drive. Continuous monitoring studies have shown that deteriorating ward patients typically demonstrate progressive tachypnoea rather than bradypnoea preceding adverse events (22).

The restricted cubic spline analysis identified a nadir of risk at a time-weighted mean RR of 16.3 breaths/min, with the range statistically indistinguishable from this nadir extending only from 13.9 to 17.4 breaths/min. Risk was already appreciably elevated at a mean rate of 20 breaths/min. This does not contradict the finding that time spent in the 20–24 breaths/min band carried little additional risk in the range-specific models, because the two analyses use different exposure metrics: the spline models a measure of central tendency, whereas the range-specific models describe how monitoring time is distributed. The two are complementary descriptions of the same exposure rather than mutual validations of one another, and we have avoided characterising them as such. Taken together they suggest that the cumulative burden of tachypnoea, rather than the occurrence of categorical threshold breaches, is the dimension most strongly associated with outcome. This parallels observations in oxygen exposure research, where time-varying patterns of hyperoxaemia and hypoxaemia show stronger associations with outcomes than single measurements (23).

These findings have potential implications for ICU monitoring strategies. Current vital sign surveillance in most ICUs relies on intermittent manual measurements, typically performed every 1–4 h depending on patient acuity. Analogous to time-in-range metrics transforming glycemic management in diabetes (24), incorporating temporal integration of RR patterns could enhance risk stratification in critically ill patients. Continuous wireless monitoring systems have demonstrated feasibility in reducing unplanned ICU transfers and rapid response team activations on general wards (25, 26). Extending such approaches to incorporate RR exposure metrics—capturing both intensity and duration—could complement existing early warning systems (25, 27). Additionally, respiratory rate variability, which reflects the complexity of respiratory control, has shown prognostic value in predicting clinical deterioration (9, 10), suggesting that both mean RR exposure and variability patterns merit clinical attention.

The consistency of our findings across three geographically and clinically diverse databases strengthens confidence in their generalisability. HiRID, MIMIC-IV and eICU represent distinct healthcare systems with different case mixes, admission practices and treatment protocols, and they differ in outcome definition (in-hospital vs. 28-day mortality), in analytical approach (Cox vs. logistic regression) and in how respiratory rate is recorded. Respiratory rate values in MIMIC-IV and eICU are almost exclusively integers recorded by nursing staff (>99.99% and 100% of observations respectively), with an over-representation of 16, 18 and 20 breaths/min of 26%–40% relative to neighbouring values, whereas HiRID provides continuously monitored values of which only 24% are integers (Supplementary Figure S6). Because outcome definitions and models were not harmonised in the primary analyses, these analyses constitute an assessment of transferability under differing outcomes rather than external validation in the predictive-modelling sense, and the concordance we observed should be interpreted accordingly. That the direction and gradient of the tachypnoea-mortality association were nonetheless reproduced under such heterogeneous conditions argues against the findings being an artefact of any single database or recording practice. Moreover, when the outcome was harmonised by relating respiratory-rate exposure to in-hospital mortality in MIMIC-IV as well, using the same model form as in eICU, the associations were unchanged (Supplementary Table S10), so the concordance we report does not rest on the choice of outcome.

Several limitations warrant consideration. First, although we adjusted for ventilation status and for sedative and opioid exposure and stratified by both, respiratory rate in ventilated patients is partly determined by ventilator settings, and residual misclassification remains possible; in eICU, invasive ventilation could be established only as a binary indicator for the exposure window and sedative exposure could not be reconstructed, so the eICU stratification is coarser than that in MIMIC-IV. Second, we could not account for specific interventions targeting RR abnormalities, such as adjustments in ventilator settings or sedation management. Third, heterogeneity in RR recording practices across institutions may introduce measurement error that biases effect estimates towards the null; the digit preference documented above is one manifestation of this. Fourth, diagnostic codes used for confounder adjustment are assigned at hospital discharge and therefore do not strictly precede the exposure window. Fifth, lactate was missing in 40.6% of MIMIC-IV and 72.6% of eICU patients within the first 24 h; multiple imputation gave results essentially identical to median imputation, with a fraction of missing information below 0.011. Sixth, the HiRID cohort excluded patients with monitoring gaps exceeding 6 h, who had a lower in-hospital mortality (3.1% vs. 10.2%; Supplementary Table S6); inverse-probability weighting to the full cohort left the exposure-outcome associations essentially unchanged, but the HiRID results are best regarded as applying to densely monitored patients. Finally, residual confounding remains possible despite extensive adjustment, and the observational design does not permit causal inference.

From a clinical perspective, these findings suggest that the cumulative burden of tachypnoea, and not only instantaneous values, carries prognostic information that is available at no additional cost from data already collected at the bedside. Because respiratory rate in critically ill patients is substantially determined by ventilator settings, sedation and the underlying disease process, we do not propose it as an independently modifiable therapeutic target. Rather, our findings support its use as a monitoring and early-warning signal, in which sustained deviation is treated as an indication to reassess the patient. Whether surveillance strategies incorporating exposure duration improve outcomes requires evaluation in prospective studies.

## Conclusion

This multi-database investigation identified an asymmetric, non-linear relationship between respiratory rate and mortality in critically ill patients, with the lowest risk at approximately 16–17 breaths/min. A greater cumulative burden of tachypnoea during the first 24 h of ICU admission was consistently associated with higher mortality across three databases, and this association was robust to controls for reverse causation, mechanical ventilation, sedation and extensive confounding. The apparent association between bradypnoea and mortality was not robust to these controls and is likely to reflect terminal-phase respiratory depression. Assessment of respiratory rate should integrate both magnitude and duration rather than relying exclusively on single-point observations, and abnormal respiratory rate is best interpreted as a monitoring and early-warning signal rather than as a therapeutic target.

## Data Availability

This study used data from the Medical Information Mart for Intensive Care IV (MIMIC-IV), the eICU Collaborative Research Database (eICU-CRD), and the High Time-Resolution ICU Dataset (HiRID). These datasets are publicly available to credentialed researchers; access requires completion of the requisite training and approval from the respective database administrators. The analysis code and derived summary data supporting the findings are available from the corresponding authors on reasonable request.
