# Supplementary material for: Temporal patterns of respiratory rate exposure and mortality in critically ill patients: a retrospective multi-database cohort study
Source: Front Med (Lausanne). 2026 Sep 30;13:1900968. doi: 10.3389/fmed.2026.1900968 (PMC13661918; doi:10.3389/fmed.2026.1900968)
Supplement: Supplementary file 1 [file Datasheet1.docx]

**Supplementary Material**

**Supplementary Tables**

**Table S1. Missing Data and Imputation Methods Across Three ICU Databases**

| **Variable** | **HiRID Datasets (n = 11,717)** | | | **MIMIC Datasets (n = 53,886)** | | | **eICU Datasets (n = 94,472)** | | |
| --- | --- | --- | --- | --- | --- | --- | --- | --- | --- |
|  | **Imputation Method** | **Imputation Value** | **Missing Percentage (%)** | **Imputation Method** | **Imputation Value** | **Missing Percentage (%)** | **Imputation Method** | **Imputation Value** | **Missing Percentage (%)** |
| Age | / | / | 0.00 | / | / | 0.00 | / | / | 0.00 |
| Sex | / | / | 0.00 | / | / | 0.00 | / | / | 0.00 |
| Hb | Median | 9.20 | 11.05 | Median | 10.10 | 1.91 | Median | 10.70 | 10.43 |
| WBC | Median | 12.1 | 2.94 | Median | 12.00 | 1.94 | Median | 11.20 | 11.24 |
| PLT | Median | 147 | 3.37 | Median | 175 | 1.93 | Median | 184 | 11.45 |
| Lactate | Median | 2.20 | 5.87 | Median | 2.10 | 40.64 | Median | 1.90 | 72.60 |
| Creatinine | Median | 0.97 | 4.95 | Median | 1.00 | 1.49 | Median | 1.01 | 8.41 |
| SOFA | / | / | 0.00 | / | / | 0.00 | / | / | 0.00 |

Missing values for continuous variables were imputed using the median value of the observed data. Variables marked with '/' indicate either no missing data or that the variable was not available in that particular database.

For MIMIC-IV and eICU, imputation values and missing percentages refer to the extreme value recorded within the first 24 hours after ICU admission, matching the covariate definition used in all adjusted models. For HiRID, which contributed only the descriptive intensity-duration analysis and no adjusted model, values refer to the extreme recorded over the ICU stay. Abbreviations: Hb, hemoglobin; WBC, white blood cell count; PLT, platelet count; SOFA, Sequential Organ Failure Assessment.

**Table S2. Baseline characteristics of the study population stratified by survival status in MIMIC-IV and eICU Databases**

| **Characteristic** | **MIMIC-IV Database (n=53,886)** | | | **eICU Database (n=94,472)** | | |
| --- | --- | --- | --- | --- | --- | --- |
|  | **Survivors (n=47,089)** | **Non-survivors (n=6,797)** | **P value** | **Survivors (n=85,652)** | **Non-survivors (n=8,820)** | **P value** |
| Age, years | 66.00 (54.00, 76.00) | 74.00 (62.00, 84.00) | <0.001 | 65.00 (53.00, 76.00) | 71.00 (60.00, 81.00) | <0.001 |
| Male sex, n (%) | 27044 (57.4) | 3659 (53.8) | <0.001 | 46507 (54.3) | 4759 (54.0) | 0.548 |
| Diabetes, n (%) | 13552 (28.8) | 2037 (30.0) | 0.045 | 24077 (28.1) | 2612 (29.6) | 0.003 |
| Hypertension, n (%) | 30135 (64.0) | 4560 (67.1) | <0.001 | 45262 (52.8) | 4961 (56.2) | <0.001 |
| Renal dysfunction, n (%) | 16064 (34.1) | 4315 (63.5) | <0.001 | 17931 (20.9) | 3450 (39.1) | <0.001 |
| COPD, n (%) | 5450 (11.6) | 1169 (17.2) | <0.001 | 13262 (15.5) | 1909 (21.6) | <0.001 |
| SOFA score | 7.00 (5.00, 9.00) | 9.00 (6.00, 12.00) | <0.001 | 2.00 (1.00, 5.00) | 6.00 (3.00, 10.00) | <0.001 |
| Minimum hemoglobin, g/dL | 10.20 (8.60, 11.90) | 9.70 (8.10, 11.30) | <0.001 | 10.70 (9.30, 12.30) | 10.30 (8.60, 11.70) | <0.001 |
| Maximum WBC, 10^9/L | 12.00 (8.60, 16.10) | 13.00 (9.30, 18.40) | <0.001 | 11.20 (8.40, 14.40) | 13.00 (9.70, 19.00) | <0.001 |
| Minimum platelet, 10^9/L | 175.00 (127.00, 235.00) | 172.00 (107.00, 239.00) | <0.001 | 184.00 (144.00, 232.00) | 179.00 (113.00, 228.00) | <0.001 |
| Maximum lactate, mmol/L | 2.10 (1.90, 2.30) | 2.10 (1.80, 3.30) | <0.001 | 1.90 (1.90, 1.90) | 1.90 (1.90, 3.20) | <0.001 |
| Maximum creatinine, mg/dL | 0.90 (0.70, 1.30) | 1.30 (0.90, 2.20) | <0.001 | 1.01 (0.77, 1.40) | 1.42 (0.98, 2.40) | <0.001 |

Values are presented as median (interquartile range) for continuous variables and number (percentage) for categorical variables. P values were calculated using the Wilcoxon rank-sum test for continuous variables and the chi-square test for categorical variables. Laboratory values are the extreme recorded within the first 24 hours after ICU admission, matching the covariate definition used in all adjusted models, and missing values were imputed with the median (Table S1); the narrow interquartile range for lactate reflects the high proportion imputed. MIMIC-IV: Medical Information Mart for Intensive Care IV; eICU: eICU Collaborative Research Database.

**Table S3. Associations stratified by mechanical ventilation and by sedative or opioid exposure (MIMIC-IV, landmark design)**

|  | **Whole cohort** | | | **Stratified analysis** | | | |
| --- | --- | --- | --- | --- | --- | --- | --- |
| **RR range** | **Fully adjusted** | **+ Ventilation & sedation** | **95% CI** | **Mechanical ventilation** | **Spontaneous breathing** | **Sedative/opioid exposed** | **No sedative/opioid** |
| <10 bpm | 0.9897 | 0.9896 | (0.9841-0.9951) | 0.9835 (0.9741-0.9930) | 0.9944 (0.9877-1.0010) | 0.9889 (0.9825-0.9955) | 0.9919 (0.9819-1.0019) |
| 10-12 bpm | 0.9830 | 0.9831 | (0.9787-0.9875) | 0.9667 (0.9576-0.9759) | 0.9893 (0.9844-0.9942) | 0.9795 (0.9738-0.9852) | 0.9858 (0.9790-0.9926) |
| 12-20 bpm | 0.9899 | 0.9897 | (0.9889-0.9906) | 0.9895 (0.9880-0.9911) | 0.9904 (0.9893-0.9914) | 0.9884 (0.9873-0.9896) | 0.9913 (0.9899-0.9926) |
| 20-24 bpm | 1.0025 | 1.0025 | (1.0013-1.0038) | 1.0050 (1.0029-1.0070) | 1.0010 (0.9994-1.0026) | 1.0039 (1.0023-1.0055) | 1.0010 (0.9989-1.0031) |
| 24-27 bpm | 1.0102 | 1.0102 | (1.0086-1.0117) | 1.0103 (1.0076-1.0130) | 1.0097 (1.0078-1.0116) | 1.0104 (1.0085-1.0123) | 1.0103 (1.0076-1.0129) |
| 27-30 bpm | 1.0158 | 1.0158 | (1.0139-1.0178) | 1.0151 (1.0116-1.0186) | 1.0153 (1.0129-1.0177) | 1.0169 (1.0144-1.0194) | 1.0148 (1.0118-1.0179) |
| 30-35 bpm | 1.0165 | 1.0165 | (1.0149-1.0182) | 1.0159 (1.0128-1.0191) | 1.0150 (1.0130-1.0171) | 1.0162 (1.0141-1.0183) | 1.0161 (1.0132-1.0189) |
| >35 bpm | 1.0214 | 1.0215 | (1.0187-1.0244) | 1.0247 (1.0194-1.0300) | 1.0196 (1.0162-1.0230) | 1.0205 (1.0166-1.0245) | 1.0223 (1.0182-1.0265) |

Hazard ratios for 28-day mortality per 1 percentage-point increase in time spent in the range. The fully adjusted model here uses the expanded confounder set of Model 7 in Table S4, which is why its estimates differ slightly from the Model 5 estimates of Table 2. Subgroup sizes: mechanical ventilation n = 15,741 (2,146 deaths); spontaneous breathing n = 38,135 (4,650); sedative or opioid exposed n = 29,350 (3,847); not exposed n = 24,526 (2,949).

Sedatives comprised propofol, midazolam, lorazepam, dexmedetomidine and ketamine; opioids comprised fentanyl, hydromorphone and morphine, all ascertained within the first 24 hours after ICU admission. Sedative use was recorded in 91.1% of ventilated and 24.8% of non-ventilated patients.

Adding ventilation status and sedative or opioid exposure to the fully adjusted model changed no hazard ratio by more than 0.0002 (largest change 0.00013). See also Figure S8.

**Table S4. Effect of expanded confounder adjustment (MIMIC-IV, landmark design)**

| **RR range** | **Model 5** | **Model 6** | **Model 7** | **95% CI** | **P** |
| --- | --- | --- | --- | --- | --- |
| <10 bpm | 0.9899 | 0.9892 | 0.9897 | (0.9843-0.9952) | <0.001 |
| 10-12 bpm | 0.9814 | 0.9813 | 0.9830 | (0.9787-0.9874) | <0.001 |
| 12-20 bpm | 0.9878 | 0.9891 | 0.9899 | (0.9890-0.9907) | <0.001 |
| 20-24 bpm | 1.0033 | 1.0030 | 1.0025 | (1.0013-1.0038) | <0.001 |
| 24-27 bpm | 1.0125 | 1.0112 | 1.0102 | (1.0086-1.0117) | <0.001 |
| 27-30 bpm | 1.0191 | 1.0172 | 1.0158 | (1.0138-1.0177) | <0.001 |
| 30-35 bpm | 1.0190 | 1.0181 | 1.0165 | (1.0148-1.0182) | <0.001 |
| >35 bpm | 1.0256 | 1.0231 | 1.0214 | (1.0185-1.0242) | <0.001 |

Model 5: the fully adjusted model of Table 2. Model 6: Model 5 plus Charlson comorbidity index, congestive heart failure, liver disease, malignancy, AIDS, Sepsis-3, do-not-resuscitate status and vasoactive agent use. Model 7: Model 6 plus acute respiratory distress syndrome and pneumonia diagnoses.

All additional covariates were ascertained within the first 24 hours after ICU admission, except diagnostic codes, which are assigned at hospital discharge. n = 53,876; 6,796 deaths (ten patients of the 53,886-patient cohort had non-positive laboratory values and could not enter the adjusted models).

Directions, gradients and statistical significance were unchanged. Effect sizes for the 12-20 breaths/min range and for all tachypnoeic ranges attenuated by 13-23% after full adjustment; the 10-12 breaths/min range attenuated by 8.7% and the <10 breaths/min range changed by 2.2% in the opposite direction.

**Table S5. Statistical sensitivity analyses (MIMIC-IV, landmark design)**

| **RR range** | **Median imputation** | **Multiple imputation (95% CI)** | **FMI** | **Bonferroni P** | **FDR P** | **PH test P** | **Early HR** | **Late HR** |
| --- | --- | --- | --- | --- | --- | --- | --- | --- |
| <10 bpm | 0.9899 | 0.9902 (0.9847-0.9958) | 0.001 | 0.017 | <0.001 | 0.908 | 0.9894 | 0.9907 |
| 10-12 bpm | 0.9814 | 0.9816 (0.9772-0.9861) | 0.002 | <0.001 | <0.001 | 0.020 | 0.9719 | 0.9882 |
| 12-20 bpm | 0.9878 | 0.9880 (0.9871-0.9889) | 0.006 | <0.001 | <0.001 | <0.001 | 0.9851 | 0.9899 |
| 20-24 bpm | 1.0033 | 1.0032 (1.0019-1.0045) | 0.003 | <0.001 | <0.001 | 0.690 | 1.0040 | 1.0028 |
| 24-27 bpm | 1.0125 | 1.0123 (1.0108-1.0139) | 0.006 | <0.001 | <0.001 | 0.003 | 1.0147 | 1.0106 |
| 27-30 bpm | 1.0191 | 1.0189 (1.0170-1.0208) | 0.011 | <0.001 | <0.001 | 0.156 | 1.0198 | 1.0186 |
| 30-35 bpm | 1.0190 | 1.0186 (1.0170-1.0202) | 0.003 | <0.001 | <0.001 | 0.080 | 1.0199 | 1.0177 |
| >35 bpm | 1.0256 | 1.0255 (1.0227-1.0282) | 0.002 | <0.001 | <0.001 | 0.359 | 1.0271 | 1.0236 |

Multiple imputation used chained equations with m = 5 imputations and Rubin's rules; the imputation model included all covariates, all eight exposure variables, the event indicator and the Nelson-Aalen cumulative hazard. FMI, fraction of missing information.

Bonferroni and Benjamini-Hochberg corrections were applied across 40 tests (8 ranges x 5 models); 39 of 40 remained significant under both.

The proportional hazards assumption was assessed with scaled Schoenfeld residuals. Early and late hazard ratios refer to days 0-6 and 6-27 after the landmark; all eight ranges attenuated towards the null over follow-up. See Figure S7.

**Table S6. Baseline characteristics of patients included in and excluded from the HiRID analysis by the monitoring-gap rule**

| **Characteristic** | **Included (n = 11,717)** | **Excluded (n = 5,044)** | **P** | **SMD** |
| --- | --- | --- | --- | --- |
| Age, years | 65.0 (55.0, 75.0) | 65.0 (50.0, 75.0) | <0.001 | 0.21 |
| ICU length of stay, h | 46.2 (32.2, 82.2) | 87.3 (46.0, 182.6) | <0.001 | -0.595 |
| Minimum haemoglobin, g/L | 92.0 (80.0, 109.0) | 89.0 (77.0, 107.0) | <0.001 | 0.1 |
| Maximum WBC, G/L | 12.1 (9.3, 16.0) | 12.5 (9.6, 16.9) | <0.001 | -0.096 |
| Minimum platelet, G/L | 147.0 (96.0, 205.0) | 161.0 (104.0, 219.0) | <0.001 | -0.103 |
| Maximum lactate, mmol/L | 2.2 (1.4, 3.7) | 2.0 (1.3, 3.1) | <0.001 | 0.152 |
| Maximum creatinine, umol/L | 86.0 (65.0, 135.0) | 77.0 (60.0, 117.0) | <0.001 | 0.082 |
| Minimum MAP, mmHg | 40.0 (0.0, 52.0) | 12.0 (0.0, 52.0) | <0.001 | 0.197 |
| GCS total | 12.0 (6.0, 14.0) | 12.0 (7.0, 14.0) | <0.001 | 0.025 |
| Maximum bilirubin, umol/L | 13.0 (8.0, 25.0) | 13.0 (8.0, 26.0) | 0.872 | -0.041 |
| In-hospital mortality, n (%) | 1,198 (10.2) | 154 (3.1) | <0.001 | 0.291 |

Values are median (interquartile range) unless otherwise indicated. SMD, standardised mean difference; an absolute value below 0.10 is conventionally regarded as negligible imbalance.

The monitoring-gap rule excluded 5,044 of the 16,761 patients meeting the length-of-stay and respiratory-rate criteria (30.1%). The remaining exclusions from the original 33,905 admissions were due to the conventional criterion of an ICU stay longer than 24 hours (17,139 patients) and absence of respiratory-rate monitoring (5 patients).

Excluded patients had a lower in-hospital mortality (3.1% versus 10.2%) and a longer ICU stay. Inverse-probability weighting to the full cohort left the exposure-outcome associations essentially unchanged (correlation of cell-level event-rate ratios 0.9965).

**Table S7. Number of exposure events contributing to each cell of the intensity-duration heatmap (HiRID)**

| **RR band (breaths/min)** | **0 min** | **30 min** | **60 min** | **90 min** | **120 min** | **Minimum** | **Cells <20 events** |
| --- | --- | --- | --- | --- | --- | --- | --- |
| 5-7 | 1,420,714 | 71 | 28 | 18 | 15 | 15 | 12 |
| 7-9 | 140,041 | 353 | 154 | 101 | 82 | 82 | 0 |
| 9-11 | 359,558 | 2,229 | 1,200 | 809 | 598 | 598 | 0 |
| 11-13 | 708,523 | 4,009 | 2,195 | 1,515 | 1,139 | 1,139 | 0 |
| 13-15 | 899,135 | 3,636 | 1,901 | 1,295 | 935 | 935 | 0 |
| 15-17 | 885,241 | 3,074 | 1,452 | 907 | 640 | 640 | 0 |
| 17-19 | 748,860 | 1,326 | 575 | 377 | 268 | 268 | 0 |
| 19-21 | 645,285 | 809 | 273 | 161 | 104 | 104 | 0 |
| 21-23 | 498,409 | 436 | 166 | 109 | 82 | 82 | 0 |
| 23-25 | 411,326 | 368 | 129 | 79 | 56 | 56 | 0 |
| 25-27 | 329,086 | 325 | 140 | 85 | 54 | 54 | 0 |
| 27-29 | 227,417 | 260 | 127 | 76 | 50 | 50 | 0 |
| 29-31 | 171,635 | 256 | 140 | 89 | 62 | 62 | 0 |
| 31-33 | 118,087 | 125 | 67 | 46 | 38 | 38 | 0 |
| 33-35 | 79,907 | 183 | 94 | 61 | 40 | 40 | 0 |

Counts are shown at selected duration thresholds; the full 15 x 41 grid is displayed in Figure S3. Median 284 events per cell (range 15 to 1,420,714).

Because a single sustained episode is counted at every duration threshold it exceeds, counts across adjacent duration columns are nested and not independent.

Sustained bradypnoea was rare at the patient level: a continuous episode in the 5-7 breaths/min band lasting 87 minutes or longer occurred in 13 patients (7 non-survivors), and one lasting 6 minutes or longer in 291 patients (93 non-survivors); the corresponding figures for the 7-9 breaths/min band are 64 (29) and 923 (160). All 12 cells containing fewer than 20 events lie in the 5-7 breaths/min band at durations of 87 minutes or longer.

**Table S8. Compositional sensitivity analysis: separate versus reference-category models (MIMIC-IV, landmark design)**

| **RR range** | **Separate models** | **Reference-category model** | **95% CI** | **P** | **VIF** |
| --- | --- | --- | --- | --- | --- |
| <10 bpm | 0.9899 | 1.0063 | (1.0006-1.0121) | 0.030 | 1.19 |
| 10-12 bpm | 0.9814 | 1.0025 | (0.9978-1.0073) | 0.300 | 1.32 |
| 20-24 bpm | 1.0033 | 1.0076 | (1.0061-1.0090) | <0.001 | 1.19 |
| 24-27 bpm | 1.0125 | 1.0100 | (1.0083-1.0118) | <0.001 | 1.23 |
| 27-30 bpm | 1.0191 | 1.0127 | (1.0104-1.0150) | <0.001 | 1.34 |
| 30-35 bpm | 1.0190 | 1.0166 | (1.0147-1.0186) | <0.001 | 1.37 |
| >35 bpm | 1.0256 | 1.0204 | (1.0171-1.0237) | <0.001 | 1.16 |

In the separate models each interval is entered alone, so its coefficient reflects increasing time in that interval with the remaining time redistributed as observed. In the reference-category model the seven remaining proportions are entered simultaneously with 12-20 breaths/min omitted, so each coefficient reflects reallocating one percentage point of monitoring time from the optimal range to the interval of interest.

The maximum variance inflation factor was 1.37, confirming that omitting one component removes the collinearity inherent in a closed composition.

Associations for the tachypnoeic intervals were consistent across both specifications, whereas the apparent protective associations of the low respiratory-rate intervals in the separate models reversed under the reference-category specification.

**Table S9. Competing-risk analysis in eICU (landmark design)**

| **RR range** | **Logistic OR** | **Cause-specific HR** | **Subdistribution HR** | **95% CI** | **P** |
| --- | --- | --- | --- | --- | --- |
| <10 bpm | 0.995 | 0.9965 | 0.9970 | (0.9935-1.0006) | 0.101 |
| 10-12 bpm | 0.983 | 0.9878 | 0.9844 | (0.9808-0.9879) | <0.001 |
| 12-20 bpm | 0.991 | 0.9932 | 0.9913 | (0.9906-0.9919) | <0.001 |
| 20-24 bpm | 1.000 | 0.9995 | 0.9996 | (0.9987-1.0006) | 0.430 |
| 24-27 bpm | 1.011 | 1.0075 | 1.0094 | (1.0083-1.0105) | <0.001 |
| 27-30 bpm | 1.017 | 1.0119 | 1.0143 | (1.0128-1.0157) | <0.001 |
| 30-35 bpm | 1.019 | 1.0098 | 1.0132 | (1.0119-1.0144) | <0.001 |
| >35 bpm | 1.020 | 1.0128 | 1.0162 | (1.0146-1.0177) | <0.001 |

Discharge alive constitutes a competing event for in-hospital mortality. n = 93,815 (8,803 in-hospital deaths, 85,012 discharged alive). Because follow-up was complete, the Fine-Gray subdistribution model is equivalent to a Cox model in which patients experiencing the competing event remain in the risk set indefinitely.

This analysis was not applicable to MIMIC-IV, in which the outcome was all-cause 28-day mortality including deaths after discharge, and all survivors were administratively censored at 28 days.

Estimates from the logistic, cause-specific and subdistribution models were consistent in direction and magnitude.

**Table S10. Harmonised-outcome analysis: in-hospital mortality modelled by logistic regression in both MIMIC-IV and eICU (landmark design)**

| **RR range** | **MIMIC-IV OR (95% CI)** | **P** | **eICU OR (95% CI)** | **P** |
| --- | --- | --- | --- | --- |
| <10 bpm | 0.989 (0.982-0.996) | 0.001 | 0.995 (0.991-0.999) | 0.025 |
| 10-12 bpm | 0.978 (0.973-0.984) | <0.001 | 0.983 (0.980-0.987) | <0.001 |
| 12-20 bpm | 0.985 (0.984-0.986) | <0.001 | 0.991 (0.990-0.991) | <0.001 |
| 20-24 bpm | 1.004 (1.002-1.006) | <0.001 | 1.000 (0.999-1.001) | 0.726 |
| 24-27 bpm | 1.015 (1.013-1.017) | <0.001 | 1.011 (1.010-1.013) | <0.001 |
| 27-30 bpm | 1.025 (1.022-1.028) | <0.001 | 1.017 (1.015-1.019) | <0.001 |
| 30-35 bpm | 1.027 (1.024-1.029) | <0.001 | 1.019 (1.017-1.021) | <0.001 |
| >35 bpm | 1.034 (1.030-1.038) | <0.001 | 1.020 (1.017-1.022) | <0.001 |

Both databases were analysed with the same outcome (in-hospital mortality), the same model (multivariable logistic regression), the same exposure window (the first 24 hours after ICU admission) and the same covariate set (age, sex, SOFA score, first-24-hour laboratory values, diabetes, hypertension, renal dysfunction and COPD). Odds ratios are per 1 percentage-point increase in the proportion of monitoring time spent in the range.

MIMIC-IV: n = 53,876, 5,277 in-hospital deaths; in-hospital mortality was obtained from the hospital discharge disposition recorded in the admissions table. eICU: n = 94,472, 8,820 in-hospital deaths.

Directions and gradients were concordant across the two databases under a single harmonised specification, indicating that the agreement reported in the main text does not depend on the choice of outcome or model form. Cluster-robust confidence intervals for eICU are given in Table 3.

**Table S11. Associations stratified by invasive mechanical ventilation in eICU (landmark design)**

| **RR range** | **Invasive mechanical ventilation** | **No invasive ventilation** |
| --- | --- | --- |
| <10 bpm | 0.9958 (0.9909-1.0006) | 0.9920 (0.9842-0.9999) |
| 10-12 bpm | 0.9831 (0.9783-0.9879) | 0.9844 (0.9784-0.9905) |
| 12-20 bpm | 0.9927 (0.9917-0.9937) | 0.9856 (0.9844-0.9868) |
| 20-24 bpm | 1.0004 (0.9990-1.0018) | 1.0002 (0.9986-1.0019) |
| 24-27 bpm | 1.0098 (1.0079-1.0116) | 1.0142 (1.0122-1.0163) |
| 27-30 bpm | 1.0156 (1.0129-1.0184) | 1.0203 (1.0178-1.0228) |
| 30-35 bpm | 1.0150 (1.0124-1.0175) | 1.0242 (1.0218-1.0266) |
| >35 bpm | 1.0170 (1.0136-1.0204) | 1.0229 (1.0202-1.0256) |

Odds ratios for in-hospital mortality per 1 percentage-point increase in the proportion of the first 24 hours spent in the range, adjusted for age, sex, SOFA score, first-24-hour laboratory values and comorbidities.

Invasive mechanical ventilation during the first 24 hours after ICU admission was defined as any of the following: a care-plan entry in the Ventilation group beginning with "Ventilated" (28,481 patients); a treatment entry under "pulmonary|ventilation and oxygenation|mechanical ventilation" (22,618); or an invasive artificial airway recorded in the respiratory-care table, that is an oral or nasal endotracheal tube, tracheostomy, double-lumen tube or cricothyrotomy (4,867). Non-invasive ventilation entries were excluded from all three sources. The composite identified 29,524 of 94,472 patients (31.3%), was defined for every patient in the cohort, and gives a prevalence comparable with the 29.2% observed in MIMIC-IV; 22,614 patients (76.6% of those classified as ventilated) were identified by at least two of the three sources.

The comparison group comprises patients without invasive ventilation, of whom 7,565 (11.6%) received non-invasive ventilation during the same window. The airway field of the respiratory-care table is unpopulated for most records and identifies only 5.2% of patients on its own; it was therefore used only as one component of the composite and not as a stand-alone definition. Associations for the tachypnoeic ranges were present in both strata with the same direction and gradient, as in MIMIC-IV (Supplementary Table S3), indicating that the findings are not an artefact of ventilator-set respiratory rates.

**Supplementary Figures**

**Figure S1. Patient selection flowchart for the MIMIC-IV cohort.**

Of 94,458 adult ICU admissions, 40,530 were excluded for an ICU stay shorter than 24 hours, age under 18 years or a non-first ICU admission, and 42 for absence of respiratory-rate monitoring, leaving 53,886 patients (6,797 deaths within 28 days).

**Figure S2. Patient selection flowchart for the eICU cohort.**

Of 200,859 adult ICU admissions, 96,608 were excluded for an ICU stay shorter than 24 hours, age under 18 years or a non-first ICU admission, and 9,779 for absence of respiratory-rate monitoring, leaving 94,472 patients (8,820 in-hospital deaths).

**Figure S3. Number of exposure events contributing to each cell of the RR intensity-duration heatmap (HiRID).**

Each cell gives the total number of exposure events across the cohort for the corresponding respiratory-rate band and duration threshold. Counts are printed in cells containing fewer than 50 events (51 cells, 8.3%); dark red cells contain fewer than 20 events (12 cells, 2.0%) and all lie in the 5-7 breaths/min band at durations of 87 minutes or longer. Median 284 events per cell (range 15 to 1,420,714). The grid corresponds cell for cell to Figure 3, and is tabulated at selected thresholds in Supplementary Table S7.

**Figure S4. RR intensity-duration heatmap under the 24-hour landmark exposure window (HiRID).**

The heatmap of Figure 3 recomputed using only respiratory-rate data recorded during the first 24 hours after ICU admission. Because the event-rate ratio is referenced to the mean across valid cells, and that mean differs between exposure windows, absolute deviation values are not comparable between this figure and Figure 3; the panel is provided to show that the tachypnoeic gradient persists when exposure cannot incorporate information recorded shortly before death.

**Figure S5. Restricted cubic spline curve for exposure accumulated over the entire ICU stay (MIMIC-IV).**

The counterpart of Figure 5 in which the time-weighted mean respiratory rate was calculated over the whole ICU monitoring period. Covariates are identical to Figure 5 and were ascertained within the first 24 hours, so the two curves differ only in the exposure window. Splines were fitted with five knots at the 5th, 27.5th, 50th, 72.5th and 95th percentiles; the reference value is the nadir of the fitted curve (16.7 breaths/min); n = 53,876 with 6,796 deaths. The solid line is the adjusted hazard ratio and the shaded band its 95% confidence interval; the grey histogram shows the distribution of the exposure.

**Figure S6. Digit preference in recorded respiratory rate across the three databases.**

Distribution of recorded respiratory-rate values. Values in MIMIC-IV and eICU are almost exclusively integers charted by nursing staff (>99.99% and 100% of observations respectively), with 16, 18 and 20 breaths/min over-represented by 26-40% relative to neighbouring values; HiRID provides continuously monitored values, of which only 24% are integers.

**Figure S7. Scaled Schoenfeld residuals for the eight respiratory-rate ranges (MIMIC-IV, landmark design).**

Each panel plots the scaled Schoenfeld residuals against follow-up time with a spline smoother; the horizontal dashed line marks the constant coefficient from the Cox model. The proportional hazards assumption was not met for three of the eight ranges, and all eight attenuated towards the null over follow-up; period-specific hazard ratios are given in Supplementary Table S5.

**Figure S8. Associations stratified by mechanical ventilation (both databases) and by sedative or opioid exposure (MIMIC-IV only) under the landmark design.**

Panel A, MIMIC-IV: hazard ratios for 28-day mortality per 1 percentage-point increase in time spent in each respiratory-rate range, within each of four strata defined by mechanical ventilation and by sedative or opioid exposure; point estimates are visually indistinguishable across strata (Supplementary Table S3). Panel B, eICU: odds ratios for in-hospital mortality in the same format, stratified by invasive mechanical ventilation ascertained from care-plan, treatment and artificial-airway records (Supplementary Table S11). In both databases the gradient across the tachypnoeic ranges is present within every stratum, indicating that it is not an artefact of ventilator-set respiratory rates. Note that panels A and B are on different scales because they report hazard ratios and odds ratios for different outcomes.
